# Validation of a brief telephone battery for neurocognitive assessment of patients with pulmonary arterial hypertension

**DOI:** 10.1186/1465-9921-6-39

**Published:** 2005-04-25

**Authors:** Darren B Taichman, Jason Christie, Rosette Biester, Jennifer Mortensen, Joanne White, Sandra Kaplan, John Hansen-Flaschen, Harold I Palevsky, C Gregory Elliott, Ramona O Hopkins

**Affiliations:** 1 Pulmonary, Allergy and Critical Care Division, University of Pennsylvania School of Medicine, Philadelphia, PA, USA; 2 Physical Medicine and Rehabilitation, University of Pennsylvania School of Medicine, Philadelphia, PA, USA; 3 Department of Medicine, Pulmonary and Critical Care Divisions, University of Utah and LDS Hospital, Salt Lake City, Utah, USA; 4 Psychology Department and Neuroscience Center, Brigham Young University, Provo, Utah, USA

## Abstract

**Background:**

The effects of pulmonary arterial hypertension on brain function are not understood, despite patients' frequent complaints of cognitive difficulties. Using clinical instruments normally administered during standard in-person assessment of neurocognitive function in adults, we assembled a battery of tests designed for administration over the telephone. The purpose was to improve patient participation, facilitate repeated test administration, and reduce the cost of research on the neuropsychological consequences of acute and chronic cardiorespiratory diseases. We undertook this study to validate telephone administration of the tests.

**Methods:**

23 adults with pulmonary arterial hypertension underwent neurocognitive assessment using both standard in-person and telephone test administration, and the results of the two methods compared using interclass correlations.

**Results:**

For most of the tests in the battery, scores from the telephone assessment correlated strongly with those obtained by in-person administration of the same tests. Interclass correlations between 0.5 and 0.8 were observed for tests that assessed attention, memory, concentration/working memory, reasoning, and language/crystallized intelligence (p ≤ 0.05 for each). Interclass correlations for the Hayling Sentence Completion test of executive function approached significance (p = 0.09). All telephone tests were completed within one hour.

**Conclusion:**

Administration of this neurocognitive test battery by telephone should facilitate assessment of neuropsychological deficits among patients with pulmonary arterial hypertension living across broad geographical areas, and may be useful for monitoring changes in neurocognitive function in response to PAH-specific therapy or disease progression.

## Introduction

Pulmonary arterial hypertension (PAH) is a devastating disease characterized by progressive shortness of breath and the eventual development of life-threatening heart failure [1-4]. While its effects on cardiovascular function have been well documented, little is known about effects of this disease on other organ systems, notably the brain. Patients frequently complain of changes in memory, concentration and judgment in association with the development of cardiopulmonary symptoms[5]. Objective assessments of cognitive function, however, have not been performed.

The "gold standard" for comprehensive assessment of neurocognitive function is a comprehensive battery of individually validated tests that are administered in-person by an experienced interviewer. Comprehensive, standard testing often takes four hours or longer and may require two or more separately scheduled sessions. The experience can be stressful and fatiguing, particularly for chronically ill or physically disabled patients. The stress of additional travel to a testing site adds to the burden. Consequently, many patients decline to participate in clinical research that uses formal in-person neurocognitive testing, especially if the protocol includes repeated testing over time [6,7].

To address these concerns, we developed a focused battery of neurocognitive tests for administration over the telephone. The battery is comprised of individually validated neurocognitive test instruments appropriate for administration to adults with cardiopulmonary disease who are fluent in English and capable of communicating verbally. Telephone administration of the test battery has been evaluated for feasibility and validity in survivors of the acute respiratory distress syndrome [8]. The present study establishes the validity of telephone administration of the test battery by comparing the results of telephone testing against "gold standard" in-person administration in adult ambulatory patients with moderately to severely symptomatic PAH.

## Methods

### Study Population

Consecutive patients diagnosed with pulmonary arterial hypertension according to standard criteria [2,9] were prospectively recruited for neurocognitive testing from the Pulmonary Hypertension Clinic at LDS Hospital. Written informed consent was obtained from the patients for both the in-person and telephone neuropsychological testing. Inclusion criteria were age 18 years or older and the ability to give informed consent. Exclusion criteria included non-fluency in English, a history of major psychiatric illness (e.g. schizophrenia, schizoaffective disorder, bipolar disorder, psychoses requiring medication and/or hospitalization, and major depression requiring hospitalization), known learning disability, prior traumatic brain injury, diagnosis of dementia, cerebral vascular accident, neurologic disorder (e.g. multiple sclerosis, Huntington's Disease, etc.), prior cardiac surgery, or current alcohol or drug abuse.

Patients were recruited for neurocognitive assessment from a group who had consented to in-person testing. Sixty-seven patients were screened. Seventeen patients declined, two were excluded due to non-fluency in English, and two were medically unstable at the time of evaluation and died during the recruitment period. Of the 46 patients who consented to in-person neurocognitive testing, 25 consented to additional testing by telephone.

Patient demographic, medical and laboratory data were collected for all enrolled patients. This study was approved by the University of Pennsylvania and LDS Hospital Institutional Review Boards and conformed to institutional and federal guidelines for the protection of human subjects.

### Neurocognitive Assessment

An interdisciplinary team of neuropsychology, traumatic brain injury, rehabilitation medicine, and pulmonary disease specialists selected a battery of standardized neurocognitive tests amenable to both in-person and telephone administration. Tests were also chosen on the basis of established sensitivity in detecting impairment in patients with cardiopulmonary disorders and concomitant hypoxemia [10-13]. The cognitive domains assessed and the tests included in the battery are listed in Table 1. All neurocognitive tests included in the battery have been empirically validated and standardized [14-17] with established reliability, internal and external validity[12,14,16,18,19]. The neurocognitive tests were administered in a random sequence to minimize order effects. However, as a delay is required between the Wechsler Memory Scale-III Logical Memory I and II tests, Logical memory I (immediate recall) was the first and Logical Memory II (delay recall) the last test administered in each session.

**Table 1:** Neurocognitive Battery for Telephone Administration

COGNITIVE DOMAIN	TEST INSTRUMENT
Attention	WMS-III: Digits Forward
Concentration/Working Memory	WMS-III: Digits Backward
	WMS-III: Letter-Number Sequencing
Executive Function	Hayling Sentence Completion Test
Reasoning	WAIS-III: Similarities
Language / Crystallized Intelligence	WAIS-III Vocabulary
Memory	WMS-III: Logical Memory I & II

The in-person assessment was carried out in a private office at LDS Hospital. The identical tests were administered subsequently by telephone at a prearranged time when patients were at home and free from distraction. To minimize potential learning effects, telephone testing was performed at least 2.5 months following in-person assessment, except with one patient who was tested 57 days after in-person evaluation. A Ph.D. neuropsychologist (ROH) administered the in-person tests and a neuropsychology doctoral student administered the telephone tests with no knowledge of the results of the previous in-person testing. During both the in-person and telephone tests, patients were instructed not to write down information and to answer questions without assistance. The in-person and telephone assessments were both conducted in single sessions, and each took 35 to 45 minutes to complete.

All neuropsychological tests were scored according to the published guidelines. Each test yields a raw score that was converted into a scaled score (mean = 10; SD = 3), which was used for statistical analyses, except for Logical Memory where the raw scores are used.

### Statistical Analysis

Descriptive statistics were carried out for demographic and medical data. The neuropsychological test scores from the in-person administration were compared to telephone test scores using interclass correlations. To facilitate interpretation of significant correlations (p ≤ 0.05) and because traditional significance levels for correlations coefficients are influenced by factors such as group size, range of scores, and multiple comparisons, we used the following conservative classification: fair correlation with coefficients between 0.21 and 0.40; moderate correlation 0.41 to 0.60; substantial correlation 0.61 to 0.80; and almost perfect correlation 0.81 to 1.00 [20].

To assess potential learning effects, systematic differences between first and second administrations for each of the tests were assessed using paired sample t-tests. The differences between the in-person and telephone test results are expressed as standardized effects sizes (T2-T1 differences divided by T1 standard deviation) [21].

## Results

Twenty-five patients with pulmonary arterial hypertension were enrolled for neurocognitive evaluation using both in-person and telephone testing. All 25 patients completed in-person testing. Telephone testing could not be completed on one subject due to a non-functioning telephone line, and one subject died of progressive right heart failure. All of the remaining 23 subjects completed both the in-person and telephone assessments and were included in the validation group. Eighty-three percent (n = 19) of these subjects were women. The mean ± SD age was 49.7 ± 13.9 years (range 20 to 60 years) and the mean education level was 13.6 ± 3.0 years (range 6 to 20 years). The mean number of days between in-person and telephone testing was 121.6 (range 57 to 200 days). The etiology of PAH was: idiopathic ("primary") PAH in ten patients (43%), associated with anorexigen use in six (26%), collagen vascular disease in four (17%), congenital heart disease in two (9%) and one with portopulmonary hypertension (4%). The mean (± SD) right atrial pressure was 5.1 ± 1.8 mmHg, mean pulmonary artery pressure 52.1 ± 16.9 and pulmonary capillary wedge pressure 12.1 ± 6.2. The mean cardiac output was 5.1 ± 1.8 L/min. Demographic and medical data are shown in Table 2.

**Table 2:** Demographic and Medical Data

	**Mean ± SD**	**Range**
Gender (% female) (n = 25)	83%	
Education (years)	13.6 ± 3.0	6 to 20
Age (years)	49.7 ± 13.9	20 to 69
Time Since Diagnosis (years)	1.8 ± 1.5	0.8 to 5.3
PaO_2 _mmHg	62.6 ± 13.5	38 to 97
Most recent 6 minute walk (meters)	455 ± 132	227 to 877
New York Heart Functional Class (N)		
Class 1	0	
Class 2	3	
Class 3	20	
Class 4	0	
Supplemental Oxygen (N)		
2 Liters per minute	7	
3 Liters per minute	8	
4 Liters per minute	4	

The results of the telephone and in-person neurocognitive assessments are shown in Table 3. The correlation coefficients for the comparison between in-person and telephone testing are presented in Table 4. Interclass correlation coefficients of at least 0.54 (p < 0.05 to < 0.0001) were found for the agreement of telephone and in-person scores on tests assessing the cognitive domains of attention, memory, concentration / working memory, reasoning, and language / crystallized intelligence. An almost perfect correlation was observed in the assessment of reasoning (Similarities). Substantial correlations were found for the Digit Span, Similarities, and Vocabulary tests (.61 to .80) and moderate correlations (.41 to .60) were found for each Logical Memory immediate and delay recall. A moderate correlation (0.56) was seen with the in-person and telephone administration of the Digit-Span-backward (concentration / working memory). Only a fair correlation (0.28; p= 0.09) was found between the in-person and telephone administration of the Hayling Sentence Completion test of executive function. For Letter-Number Sequence test (concentration/working memory) scores were not correlated for the in-person and telephone tests.

**Table 3:** In-person and telephone neuropsychological test scores.

**Test**	**Mean**	**Median**	**SD**	**Range**
*Number-letter Sequencing*				
In-person	9.3	10	2.6	5 to 14
Telephone	9.3	9	2.5	5 to 15

*Logical Memory*				
Immediate Recall†				
In-person	24.4	23	5.9	14 to 33
Telephone	27.0	26	8.2	17 to 44
Delay Recall†				
In-person	18.8	18	5.4	10 to 30
Telephone	22.0	23	7.5	6 to 34

*Digit Span*				
In-person	11.6	11	3.2	6 to 18
Telephone	9.3	9	2.4	5 to 15

*Hayling Sentence Completion Test*				
In-person	5.7	6	1.1	4 to 8
Telephone	6.6	6	0.89	6 to 10

*Similarities*				
In-person	11.0	12	2.9	4 to 16
Telephone	10.9	10	3.1	5 to 16

*Vocabulary*				
In-person	10.6	11	2.6	6 to 16
Telephone	11.5	11	2.6	7 to 17

All values are scaled scores (mean = 10, standard deviation = 3) except † = raw scores.

Logical memory and Number-letter sequencing are from the WMS-III; digit span, similarities and vocabulary are from the WAIS-R.

**Table 4:** Reliability of the in-person and telephone neuropsychological test scores.

**Neuropsychological Test**	**Learning Effect (Improvement T1 to T2 expressed in SDs)**	**Intraclass Correlation**	**95% C.I.**	**p**
*Number-letter Sequencing*	.78 (0 to 3.1)	.15	-.28 to .52	0.25

*Logical Memory*				
Immediate Recall†	1.3 (0 to 5.0)	.55	-.20 to .72	0.05
Delay Recall†	1.2 (.18 to 3.4)	.54	-.24 to .82	0.05

*Digit Span*				
Forward†		.74	.41 to .90	0.0002
Backward†		.56	-.24 to .84	0.05
Both	.83 (0 to 1.9)	.63	.29 to .83	0.0006

*Hayling Sentence* *Completion Test*	.98 (0 to 3.8)	.28	-.14 to .61	0.09
*Similarities*	.65 (0 to 2.0)	.82	.62 to .92	<0.0001
*Vocabulary*	.53 (0 to 1.5)	.68	.38 to .85	0.001

All scores shown are scaled scores (mean = 10, standard deviation = 3), except † = raw scores. Logical memory and Number-letter sequencing are from the WMS-III; digit span, similarities and vocabulary are from the WAIS-R.

Stability over time was greatest for the Similarities and Vocabulary tests. The effects of learning showed that test scores tended to increase between the in-person and telephone tests, with the most improvement for verbal memory (e.g. logical memory immediate and delayed recall).

## Discussion

We found the battery of well-established neurocognitive tests to be amenable to administration by telephone and valid for the identification of neurocognitive deficits in patients with PAH. Testing was readily completed in a single, 30–60 minute session, and required neither specialized testing facilities nor travel by physically debilitated patients spread across a broad geographic area.

Our study was designed to validate the administration by telephone of a battery of neurocognitive tests against the in-person ("gold standard") performance of these same assessments. Each of the tests in the battery has been previously validated for the identification of neurocognitive deficits in various populations, including those with cardiopulmonary disease-. As such, our subjects' scores during in-person testing served as matched controls for comparison with the results obtained upon application of these same tests over the telephone.

The scores from telephone and in-person assessments correlated strongly for the majority of tests. Overall, the strengths of the correlations with in-person testing found here are comparable to those that we reported previously for the same test battery applied to ARDS survivors [8] and the correlations reported for other telephone neurocognitive test batteries [21-25]. Two items in the test battery did not correlate as well as the others. The interclass correlations for the Hayling Sentence Completion test only approached significance (p = 0.09). The in-person and telephone administrations of the Letter-Number-Sequencing component of the WMS III (a test of concentration/working memory) did not correlate well. Some subjects appeared to have difficulty discriminating phonetically similar sounds (e.g. the letters 'm' and 'n') when presented during telephone sessions; visual cues may have alleviated such issues at in-person sessions. In contrast, another component of the WMS III (Digits Backward) evaluating the same cognitive domain (concentration/working memory) had substantial correlations.

Although the correlations we found between in-person testing and subsequent telephone administration of the same test battery were moderate or higher, they were not perfect. The effects of learning or practice suggest that test scores increase between the in-person and telephone tests, with the most improvement in verbal memory. Thus, the improvement in test scores on the telephone administration of the test likely reflects practice effects. An alternative explanation for the tendency of subjects to perform better on the telephone test battery may be environmental factors. For example subjects scored higher on certain tasks when assessed at home as compared to similar tasks performed in a clinic setting [26]. Improved orientation to time and place have been found when patients were tested in their own residence [22]. Further, patients report less anxiety and prefer telephone testing compared to in-person evaluation [27]. In addition to the pragmatic advantages, telephone testing may provide a better assessment of patients' cognitive function in their normal environment. Finally, it is possible that neurocognitive function improved during the interval between the two test sessions (mean 122 days). The reason for a potential improvement in neurocognitive performance will be important in future studies that use repeated test administration to determine the effect of drugs for PAH or other interventions on neurocognitive function.

An important limitation of our study was our inability to reverse the order of administration (in-person and telephone) [28]. Our subjects were enrolled in another ongoing study, which required in-person assessments prior to enrollment in this study of telephone testing [29]. An alternative would be to repeat in-person and telephone assessments in random order following the initial interview. Such additional testing, however, might have increased the potential for learning or practice affects, and the further time and travel commitments for patients likely impacting study participation. Future studies should counterbalance the order of in-person and telephone administration. Due to pragmatic limitations the time interval between in-person and telephone testing was somewhat longer than the minimum time necessary to minimize recall and learning effects.

Telephone testing has been used successfully in the assessment of neruocognitive impairment in other patient populations. Further, the ease in application and relative low cost of telephone testing have enabled assessments in several large clinical studies of cognitive function: screening of 4,932 elderly patients for Alzheimer's Disease using the modified Mini-Mental State Examination for telephone administration [30]; cognitive function in 4,023 patients with cardiovascular risk factors [31]; 466 patients with coronary artery bypass graft surgery [32], and in a self-referred ARDS patient group [8]. In addition to neurocognitive testing, telephone-based assessments have provided accurate determinations of quality of life, medication usage, 24-hour physical activity and dietary recall [33-35].

Cognitive function has not been studied in pulmonary arterial hypertension, despite the frequent reports of problems with memory and concentration [5]. Cognitive impairments are important complications of other chronic and life-threatening illnesses, and are associated with a significantly worse prognosis [36-38]. Cognitive impairments can also profoundly reduce quality of life [39-42]. Therapies that improve physical function in PAH may have important (but as yet unknown) effects on neurocognitive function, positive or negative. For these reasons, research on neurocognitive function is warranted. The brief neurocognitive telephone test battery is valid for assessment of cognitive function in this population and provides the means to pursue further, larger studies to assess the frequency and risk factors of cognitive sequelae in patients with PAH.

## Conclusion

This study has demonstrated that scores on a battery of neurocognitive tests obtained by telephone administration correlated well with in-person testing in patients with pulmonary arterial hypertension. The strong correlations observed are comparable to previous studies that assessed in-person and telephone versions of neurocognitive tests. With minor modification, the telephone neuropsychological test battery described here provides an economical and reliable method for assessing cognitive function in patients with pulmonary arterial hypertension.

## List of Abbreviations Used

PAH: Pulmonary Arterial Hypertension

SD: Standard Deviation

ARDS: Acute Respiratory Distress Syndrome

WMD: Wechsler Memory Scale

WAIS: Wechsler Adult Intelligence Scale

## Competing Interests

The author(s) declare that they have no competing interests.

## Authors' Contributions

DBT, JHF, HIP and ROH designed and SK coordinated this study. RP and JC designed the neurocognitive testing battery. JM, JW and ROH performed and interpreted the neuropsychological assessments and ROH the statistical analysis. CGE recruited patients and assessed results. The manuscript was written by DBT, JHF and ROH and has been read and approved by all authors.
